# Putative Role of Prostaglandin Receptor in Intracerebral Hemorrhage

**DOI:** 10.3389/fneur.2012.00145

**Published:** 2012-10-22

**Authors:** Shekher Mohan, Abdullah S. Ahmad, Alexander V. Glushakov, Chase Chambers, Sylvain Doré

**Affiliations:** ^1^Department of Anesthesiology, College of Medicine, University of FloridaGainesville, FL, USA; ^2^Departments of Neurology, Psychiatry, and Neuroscience, College of Medicine, University of FloridaGainesville, FL, USA

**Keywords:** brain, stroke, inflammation, GPCR, therapy, neurodegenerative diseases

## Abstract

Each year, approximately 795,000 people experience a new or recurrent stroke. Of all strokes, 84% are ischemic, 13% are intracerebral hemorrhage (ICH) strokes, and 3% are subarachnoid hemorrhage strokes. Despite the decreased incidence of ischemic stroke, there has been no change in the incidence of hemorrhagic stroke in the last decade. ICH is a devastating disease 37–38% of patients between the ages of 45 and 64 die within 30 days. In an effort to prevent ischemic and hemorrhagic strokes we and others have been studying the role of prostaglandins and their receptors. Prostaglandins are bioactive lipids derived from the metabolism of arachidonic acid. They sustain homeostatic functions and mediate pathogenic mechanisms, including the inflammatory response. Most prostaglandins are produced from specific enzymes and act upon cells via distinct G-protein coupled receptors. The presence of multiple prostaglandin receptors cross-reactivity and coupling to different signal transduction pathways allow differentiated cells to respond to prostaglandins in a unique manner. Due to the number of prostaglandin receptors, prostaglandin-dependent signaling can function either to promote neuronal survival or injury following acute excitotoxicity, hypoxia, and stress induced by ICH. To better understand the mechanisms of neuronal survival and neurotoxicity mediated by prostaglandin receptors, it is essential to understand downstream signaling. Several groups including ours have discovered unique roles for prostaglandin receptors in rodent models of ischemic stroke, excitotoxicity, and Alzheimer disease, highlighting the emerging role of prostaglandin receptor signaling in hemorrhagic stroke with a focus on cyclic-adenosine monophosphate and calcium (Ca^2+^) signaling. We review current ICH data and discuss future directions notably on prostaglandin receptors, which may lead to the development of unique therapeutic targets against hemorrhagic stroke and brain injuries alike.

## Introduction

Stroke is a leading cause of long-term disability and accounts for one of every 18 deaths in the United States (Roger et al., 2012). More than 795,000 people experience a new or recurrent stroke in the United States each year and 10–15% experience a hemorrhagic stroke (Thom et al., 2006; Roger et al., 2012). Although the incidence of intracerebral hemorrhage (ICH) is lower than that of ischemic stroke, the mortality and disability rates are greater. It is thought that many of the deleterious effects of ICH are due to the release of blood, increased intracranial pressure, and ischemic damage to the surrounding brain tissue (Gong et al., 2001). Current measures to reduce mortality and increase functional recovery include early diagnosis, blood pressure management, hypothermia, and surgical removal of blood and clots (Adeoye and Broderick, 2010; Kollmar et al., 2010). Due to the complexity of ICH, the development of effective interventions has been challenging.

In experimental models of ICH, increased neuronal loss has been correlated with the development of localized collection of blood outside the blood vessels called hematomas (Fernandes et al., 2000). Hematomas are made up of red blood cells, in turn consisting of hemoglobin, a complex metalloprotein containing four heme molecules whose iron atoms temporarily bind oxygen molecules. When released from red blood cells, heme-iron molecules contribute to the expansion of ICH-induced brain damage (Macdonald and Weir, 1991; Hua et al., 2000). Due to the toxic role of red blood cells in ICH, we and others have been determining whether intracranial injection of blood mimics many of the hallmarks of ICH (Wang et al., 2008). Over the past years, we have been interested in the regulation of the pro-oxidant heme by the catalytic heme oxygenase enzymes and the impact of this process on ischemic stroke (Doré et al., 1999; Li et al., 2009; Zeynalov et al., 2009) and ICH (Wang et al., 2006; Wang and Doré, 2007a, 2008). Based on the importance of heme toxicity, we are interested in determining the role of blood components in hemorrhagic stroke (Namiranian et al., 2005; Wang and Doré, 2007b).

Inflammation is the immune system’s response to infection and injury, but if persistent, it can also lead to the loss of cellular and organ function. For example, the inflammatory milieu following a hemorrhagic stroke is integral to the development of secondary injury; important components of this have traditionally included the so-called “pro-inflammatory” prostaglandins (Wang and Doré, 2007b). Despite previous work on experimental ICH by others, the role of prostaglandin receptors in ICH remains to be explored. Prostaglandins are a large family of lipids enzymatically derived from arachidonic acid by the cyclooxygenase enzymes COX-1 and -2. Prostaglandins function by activating corresponding prostaglandin receptors (DP1–2, EP1–4, FP, IP, and TP). The diversity of the receptors allows prostaglandins to act on an array of cells with a wide variety of effects. In this review, we discuss the regulation of prostaglandin receptors through selective pharmacologic ligands or genetic deletion with regard to stroke with special emphasis on ICH.

## Pathophysiology and Clinical Features of ICH

Intracerebral hemorrhage is a medical emergency that requires rapid diagnosis and management to minimize neuronal loss and deterioration after ICH. Vomiting, severe headaches, increased systolic blood pressure, and coma are common symptoms of ICH and confirmation the ICH incidence is essentially achieved by neuroimaging techniques (Sarrafzadeh et al., 2003; Goldstein and Simel, 2005).

### Vasoconstriction and blood pressure

Reversible cerebral vasoconstriction is characterized by severe headaches with or without seizures and focal neurological deficits, and constriction of cerebral arteries following occurs in 7% of ICH patients, and 22% of SAH patients (Ba et al., 2012). Increase in cerebral vasoconstriction directly affects blood pressure. Elevated blood pressure (BP) at admission has been found to predict worse outcomes; early intensive BP management reduces the risk of hematoma expansion (Anderson et al., 2010). Continuous intravenous infusion of BP lowering drugs aggressively reduces systolic BP (Manawadu et al., 2010). Prostaglandin E_2_ (PGE_2_), which is synthesized in the vasculature of the brain (within neuron and glia cells), acts as a powerful endogenous pyrogenic mediator of the preoptic area by activating specific receptors (Nakamura et al., 2009). Due to elevated BP, there is an increased risk of hemorrhage in the brain. As blood leaks and collects into a hematoma, pressure builds on nearby brain tissue, reducing vital blood flow, and killing brain cells.

### Intracranial pressure and edema

Blood components such as serum may be connected to the pathogenesis of secondary brain injury after ICH (Wang et al., 2011). Inflammatory agents released from hematomas can cause the breakdown of blood-brain barrier, which is a proposed mechanism of edema (Wagner et al., 1998). Uncontrolled inflammation contributes to brain edema and prostacyclin PGI_2_ has been reported to contribute by augmenting the permeability of capillaries (Masada et al., 2001; Xi et al., 2001, 2002; Bentzer and Grande, 2004). Following experimental lipopolysaccharide (LPS)-induced edema, low-dose infusion of PGI_2_ caused further increase in intracranial pressure (Gardenfors et al., 2004). Recently, in a mouse model of subarachnoid hemorrhage (SAH), cerebral edema was reduced following the inhibition of inducible cyclooxygenase-2 (Ayer et al., 2011). In addition to inflammatory components, iron-mediated free radical damage can also contribute to secondary damage after ICH. For example, iron released from heme after the breakdown of hemoglobin accumulates in the parenchyma and has been linked to cerebral edema; thus, removal of the hematoma reduces edema formation (Wagner et al., 1999; Huang et al., 2002; Qing et al., 2009; Zhao et al., 2011). Due to the significant impact of inflammatory components like prostaglandins following ICH, we propose that prostaglandin receptors may be ideal targets to fight against ICH-induced brain edema.

### Inflammation

The major inflammatory cells that are activated and accumulate within the brain after ICH are blood-derived leukocytes, macrophages, and resident microglia. In addition, the infiltration of short-lived neutrophils following ICH contributes to blood vessel disruption, blood-brain barrier degradation, axon damage, and the glia responses that evolve after ICH (Moxon-Emre and Schlichter, 2011). Following ischemic stroke, infiltrating neutrophils play a role in exacerbating inflammation by up-regulating matrix metalloproteinase (MMP; Justicia et al., 2003). For example, a correlation between MMP-9 activity and hemorrhagic transformation of the ischemic lesion has been reported (Heo et al., 1999; Lapchak et al., 2000; Sumii and Lo, 2002; Gautier et al., 2009).

In addition to their effect on inflammation, microglial cells are increasingly being studied as the cell type responsible for the resolution of hematomas formed after ICH (Zhao et al., 2007). Data suggest that microglia activation occurs very early after the onset of ICH and persists for weeks (Hickenbottom et al., 1999; Xue and Del Bigio, 2000). However, uncontrolled microglia activation can also play a role in secondary damage following ICH (Wasserman et al., 2008). In addition to being expressed in neurons, prostaglandin receptors are expressed in microglia, astrocytes, and endothelial cells (Caggiano and Kraig, 1999; Takemiya et al., 2011). Microglia predominately express EP1 and EP2 receptors, reactive astrocytes express EP1 and DP1 receptors and endothelia cells express all four EP (EP1–4) receptors (Mohri et al., 2007; Carlson et al., 2009; Taniguchi et al., 2011a). As well as expressing receptors, microglia and astrocytes are also a source of calcium-mediated PGE_2_ in the brain (Sanzgiri et al., 1999; Zonta et al., 2003; Anrather et al., 2011; Font-Nieves et al., 2012).

Also, due to the increased permeability of the blood-brain barrier following ICH, blood components, and plasma proteins enter the brain, initiating an exacerbated inflammatory response involving glial activation, release of cytokines, chemokines, and formation of reactive oxygen species, together resulting in the breakdown of brain tissue and repair (Peeling et al., 1998; Mayne et al., 2001a,b). For example, in a rat model of ICH induced by double autologous intrastriatal blood injection, elevated levels of interleukin-1β and tumor necrosis factor-α was observed at 3 and 24 h after injection (Mayne et al., 2001b).

Growing knowledge of the pathophysiology of ICH has led to the exploration of neuroprotective strategies aiming to improve its outcomes through reduction of secondary pathological processes. Work by us and others have previously shown that prostaglandins are important agents released in response to ischemic stroke and their affect is prostaglandin receptor-dependent. Despite some recent data by our group showing that when deleted, EP1 receptor may exacerbate brain injury in the early hours after ICH by regulating microglial phagocytosis, further studies of the role prostaglandin receptors (EP1–4) on the function of cells such as microglia and neurotrophils after ICH is still needed. More information on the general role of various cell types modulating inflammation after ICH can be found in a previous review from our group (Wang and Doré, 2007b).

### Seizures

Seizures related to ICH occur in approximately 11% of patients, although those related to ischemic stroke appear in approximately 9% of patients (De Reuck, 2007). Post-stroke seizures are generally classified as early- and late-onset seizures, with most early onset seizures occurring at onset or within the first 24 h of ICH (Takasugi et al., 1985). Early onset seizures and increased risk of epilepsy are associated with hemorrhagic stroke (Burn et al., 1997; Berges et al., 2000; De Herdt et al., 2011). Using animal models of seizure, data suggest that hemolysis and deposition of iron rich compounds may play a significant role (Willmore and Triggs, 1991). It is believed that in iron-induced experimental epilepsy models, seizure activity is mediated by free radicals and membrane lipid peroxidation (Jyoti et al., 2009). The role of pro-inflammatory molecules is also likely to contribute to ICH-related seizures.

In experimental models of kainic acid-induced seizures, COX-2 and PGE_2_ levels are increased in neuronal and non-neuronal cells and blockade of COX-2 and/or PGE_2_ reduced cell death (Takemiya et al., 2006). Vascular endothelial cells in the brain produce PGE_2_ in response to excitotoxicity (Takemiya et al., 2010). When blocked, PGE_2_ receptors EP1, EP3, and EP4 and activation of EP2 receptors have anticonvulsant and neuroprotective properties in different rodent seizure models (Oliveira et al., 2008; Fischborn et al., 2010; Takemiya et al., 2011; Rehni and Singh, 2012). Nevertheless, in a mouse model of pilocarpine-induced status epilepticus consecutive inhibition of EP2 receptors after termination of seizures with antiepileptic drugs significantly reduced hippocampal neuronal injury (Jiang et al., 2012). Furthermore prostaglandin PGF_2α_ and its FP receptor have been implicated in kainic acid-induced seizures (Kim et al., 2008). Consequently, it is likely that the production of prostaglandins following ICH may contribute to the incidence and severity of the seizures.

## Role of Prostaglandin Receptors in ICH

Prostaglandins are generated from arachidonic acid, a 20-carbon unsaturated fatty acid, which is released from the bilipid layers of the plasma membrane by the action of three different phospholipases A_2_: secreted (sPLA_2_α), and cytosolic calcium-dependent (sPLA_2_α) and calcium independent (iPLA_2_α) and metabolized to PGH_2_ by cyclooxygenase enzymes, COX-1 and COX-2 (Kishimoto et al., 2010). PGH_2_ is the common substrate for a series of specific isomerase and synthase enzymes that produce prostaglandins. Prostaglandins PGE_2_, PGI_2_, PGD_2_, PGF_2α_, and TxA_2_ are generated by the action of their respective synthases: PGE synthase (cytosolic; cPGES-1, and membrane-associated; mPGES-1 and mPGES-2), prostaglandin I synthase (PGIS), prostaglandin D synthase (hematopoietic-type: H-PGDS, and lipocalin-type: L-PGDS), prostaglandin F synthase (PGFS), and thromboxane synthase (TxS). It should be noted that mPGES-1 was found to be significantly increased in the AD brains as compared to age matched controls (Chaudhry et al., 2008, 2010). Once synthesized, prostaglandins can be further metabolized or they can act on their specific G-protein coupled receptors.

### Prostaglandin receptor expression (message and protein)

Elegant work was done to measure the respective affinity of the various radioactive-labeled prostaglandin ligands to their respective receptors by expressing each of the receptors in CHO cells and performing traditional ligand binding assays (Kiriyama et al., 1997). Furthermore, specific binding of PGD_2_, PGE_2_, and PGF_2α_ were found in rat and human brain tissue (Watanabe et al., 1985). In postmortem human brains of normal subjects, [^3^H]-PGD_2_ and [^3^H]-PGE_2_ bindings were highest in the hypothalamus, amygdala, and hippocampus followed by cerebellar nuclei and the thalamus. In addition [^3^H]-PGD_2_ was found in nucleus accumbens and cerebral cortex. [^3^H]-PGF_2α_ binding was most abundant in the amygdala, cingulate cortex, cerebellar medulla, hippocampus, nucleus accumbens, midbrain, and hypothalamus (Watanabe et al., 1985). Similar regional distribution of PGE_2_ binding sites were detected in the rat brain (Matsumura et al., 1992).

### PGE_2_-EP receptor expression

PGE_2_-EP2 receptors are expressed throughout the PNS and CNS, for example, at the mRNA level, the EP1 receptor is mainly expressed in the thalamus and hypothalamus, and have been studied in neurons and microglia under different pathologic conditions (Batshake et al., 1995; Caggiano and Kraig, 1999; Kawano et al., 2006). EP2 receptor mRNA are expressed mostly in astrocytes and neurons of the cerebral cortex, striatum, hippocampus, and CA1 pyramidal neurons (Zhang and Rivest, 1999). The mRNA expressions of EP3 receptor are most abundant in the olfactory system, hippocampus, and subcortical telencephalic structures in the septal region and amygdala of the brain (Sugimoto et al., 1994; Ek et al., 2000; Nakamura et al., 2000). EP3 receptor mRNA are expressed mostly in subcortical-hypothalamic regions of the brain owing to their role in thermoregulation (Vasilache et al., 2007). The EP3 receptor has multiple splice variants differing in their C-terminal tails. In human tissue, nine mRNA and eight isoforms (EP3_I_, EP3_II_, EP3_III_, EP3_IV_, EP3_V_, EP3_IV_, EP3_e_, and EP3_f_) have been described (Kotani et al., 1995, 1997; Schmid et al., 1995). EP3 receptor variants are mostly expressed in clusters of multiple isoforms; for example, the human uterus expresses mRNA for EP3_V_ and EP3_VI_ receptor isoforms, whereas in primary keratinocytes, EP3_I_, EP3_II_, and EP3_IV_ splice variants are expressed (Kotani et al., 2000). Similar to humans, mice also have EP3 receptor alternative splicing that are well characterized EP3 isoforms (α, β, and Γ) and contain carboxyl tails of 30, 26, and 29 amino acids. EP4 receptor mRNA are expressed in the forebrain, hypothalamus, lower brainstem, and at lower levels in endothelial cells (Narumiya et al., 1999; Zhang and Rivest, 1999; Li et al., 2008). Messenger RNA of the EP1–4 receptors was detected both in the hippocampus and in the neocortex. All four immunoreactive EP receptors appeared to be detected in neurons and were also present in astrocytes, though perhaps at weaker levels (Zhu et al., 2005).

### IP, DP, FP, and TP receptor expression

IP receptor mRNA is expressed in many tissue types including spleen, thymus, aorta, coronary, pulmonary, and cerebral arteries, kidney, and in neuronal cell bodies (Oida et al., 1995). DP1 receptor mRNA is expressed in the cerebral cortex, hippocampal pyramidal layers, dentate gyrus, thalamus, choroid plexus, and leptomeninges (Oida et al., 1997). The mRNA expression of the FP receptor has been previously demonstrated in mouse brains and in brain synaptosomes of newborn pigs and in human eye tissue (Li et al., 1993; Kitanaka et al., 1994; Liang et al., 2008). FP receptor mRNA is highly expressed in rodent whole brain (www.brain-map.org; Kitanaka et al., 1994). The FP receptor is also expressed in neuronal and astrocyte cultures (Kitanaka et al., 1991, 1993; Gotoh et al., 1994). In the brain, astrocytes, oligodendrocytes, and white matter of the striatum express TP receptor mRNA (Borg et al., 1994; Kitanaka et al., 1995, 1996; Honma et al., 2006). Like the expression patterns of prostaglandin receptors, when activated, the signaling cascades that follow are also varied.

Figure 2 illustrates the overview of prostaglandin receptor-mediated signaling. Depending on a given prostaglandin receptor, we and others have found that global deletion of specific prostaglandin receptors can greatly influence stroke outcomes (Ahmad et al., 2005, 2006a, 2008, 2010a; Saleem et al., 2007a, 2009a,b,c). However, the mechanisms that mediate these outcomes following ICH have not been fully elucidated. The prostaglandin receptors can be grouped according to the downstream signaling pathways they activate.

### Prostaglandin receptors and signaling

Prostaglandins exert cellular affects through their specific receptors: PGE_2_ receptors EP1, EP2, EP3, and EP4; PGD_2_ receptors DP1 and DP2; PGF_2α_ receptor FP; PGI_2_ receptor IP; and TxA_2_ receptor TP (Narumiya et al., 1982, 1991, 1999; Ogorochi et al., 1984; Narumiya and Toda, 1985; Ito et al., 1989; Namba et al., 1992, 1994; Honda et al., 1993; Irie et al., 1993; Watabe et al., 1993; Hirata et al., 1994a, 1996; Katsuyama et al., 1994, 1995; Hasegawa et al., 1996; Ishikawa et al., 1996; Kiriyama et al., 1997; Kobayashi and Narumiya, 2002; Woodward et al., 2011). Alternative splicing of the C-terminal has generated additional isoforms of human TP (TPα, TPβ), FP (and some relatively rare splice variants, not receptor subtypes), and eight EP3 receptor isoforms (Narumiya and Fitzgerald, 2001). Both human and mouse prostaglandin receptors signal via either G_αs_ (EP2, EP4, IP, and DP1 receptors) to increase intracellular levels of cyclic-adenosine monophosphate (cAMP), or G_αq_ (EP1, EP3, and FP) to increase intracellular levels of calcium or G_αs_-proteins (EP3, DP2, and TP receptors) to increase or decrease intracellular levels of cAMP and calcium. Table 2 depicts these prostaglandin receptors and their signaling and binding properties.

In addition to cAMP-PKA dependent second messenger signaling, which activates G-protein, G-protein activation also occurs via a PKA-independent mechanism. An example of protein activated by cAMP but PKA-independent include the exchange protein directly activated by cAMP (Epac1 and 2). Epac1 and 2 function as guanine nucleotide exchange factors for the small G-protein Rap. Epac proteins are expressed throughout the body, with Epac1 specifically abundant in blood vessels, kidney, adipose tissue, CNS, ovary, and uterus, whereas Epac2 is mostly expressed in the CNS, adrenal gland, and pancreas (de Rooij et al., 1998; Kawasaki et al., 1998). Due to the abundance of GPCRs that mediate cAMP signaling, Epac proteins have many biological functions. For example, Epac regulates electrically evoked Ca^2+^ transients in response to β-adrenergic receptors, Epac2 activation induces exocytosis in human β cells, increasing the number of exocytic sites on the plasma membrane via its effects on Ca^2+^ signaling, and effects neuronal function following activation by potentiating the postsynaptic excitation currents (Kang et al., 2003; Kaneko and Takahashi, 2004; Wang et al., 2005; Cheung et al., 2006; Gekel and Neher, 2008). Furthermore, together with PKA, Epac also contributes to the regulation of neuronal differentiation, neurite outgrowth, and axon generation, implicating an Epac role in the development and maintenance of the nervous system (Christensen et al., 2003; Kiermayer et al., 2005; Shi et al., 2006; Murray and Shewan, 2008). In addition and perhaps relevant to the ICH-pathology, Epac proteins have been implicated in vascular function.

**Figure 1:**
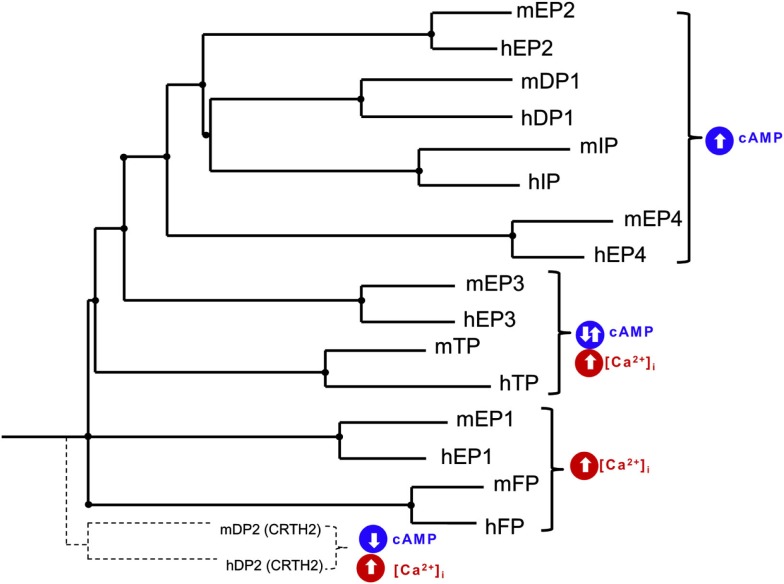
**Phylogenetic tree of human (h) and mouse (m) EP1, EP2, EP3, EP4, FP, IP, TP, DP1, and DP2 (CRTH2) receptors**. Tree was constructed using pairwise sequence alignment determined using CLUSTALW 2.1 Multiple sequence alignment software and presented as NJplot. Evolutionally conserved prostaglandin receptors can be grouped into signaling pathways: cAMP stimulating and calcium signaling.

**Figure 2:**
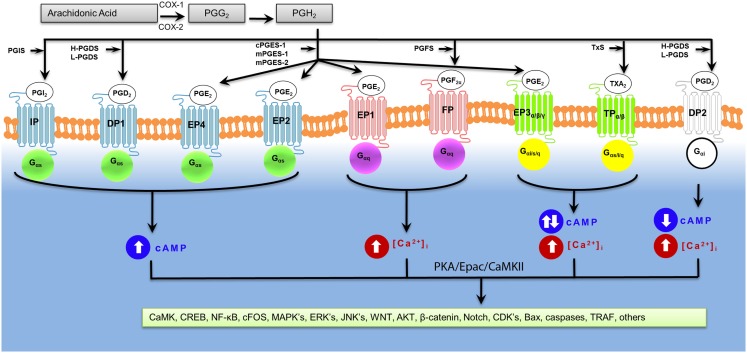
**Signal transduction mechanisms of activated prostaglandin receptors**. Prostaglandin receptors have been grouped into specific G-protein-dependent cAMP and/or Ca^2+^ signaling.

*In vivo* administration of a Epac agonist (007) inhibited vascular endothelial growth factor-induced dye leakage from mouse dermal blood vessels (Fukuhara et al., 2005). Also, 007 induced secretion of von Willebrand factor-containing Weibel–Palade bodies, which may further contribute to the regulation of vasculature homeostasis by Epac1 (Rondaij et al., 2004). Epac proteins have also been implicated in inflammation, where cAMP signaling directly controls inflammation by regulating leukocyte-mediated immune responses (Lorenowicz et al., 2007). Epac proteins are expressed in leukocytes and following the activation of Epac1 regulate the monocytes adhesion and chemotaxis (Lorenowicz et al., 2006). Epac1 also induces pathogen-mediated production of pro-inflammatory cytokines and chemokines (Gerlo et al., 2010). Due to recent reports on the role of Epacs in the brain and neurons, we hypothesis that Epacs may function to minimize neuronal death involved in ICH. Such Epac-mediated neuroprotection may occur by potentiating PKA-independent activation of the small G-protein Rap by cAMP. Mechanisms of Epac-dependent neuroprotection remain to be studied in correlation to prostaglandin receptor activation. Protein homology between human and mouse prostaglandin receptors are at best 88% (i.e., human FP receptor vs. mouse FP receptor) with little homology between receptor subtypes. A phylogenetic tree and table of the amino acid homology (% identity) between human and mouse prostaglandin receptors is illustrated in Table 1 and Figure 1, respectively. Due to the mechanistic nature of prostaglandin receptors, evolutionary relationships among human and mouse prostaglandin receptors can be broadly grouped into two signaling pathways: essentially leading to cAMP or calcium signaling cascade. Table 2 and Figure 2 illustrate signal transduction mechanisms activated when prostaglandin receptor specific agonist bind. Prostaglandin receptors have been grouped into specific G-protein-dependent cAMP and/or Ca^2+^ signaling. Prostaglandin receptors are expressed throughout the peripheral and central nervous system and therefore play an important role in the physiologic response following injury.

**Table 1:** **Amino acid homology (% identity) between human (h) and mouse (m) prostaglandin receptors**.

Receptor	hEP1	hEP2	hEP3	hEP4	hFP	hIP	hTP	hDP1	hDP2
mEP1	**85**	28	30	23	34	23	29	22	12
mEP2	25	**85**	25	32	18	38	23	41	11
mEP3	33	24	**79**	25	29	27	30	23	15
mEP4	26	23	23	**88**	20	34	22	29	13
mFP	34	20	30	19	**88**	21	34	20	14
mIP	23	40	25	25	20	**79**	25	37	15
mTP	34	23	28	24	34	27	**74**	24	14
mDP1	24	41	22	29	18	36	23	**71**	14
mDP2 (CRTH2)	24	41	22	29	18	36	23	12	**79**

*EP1 (Accession no. h: NP_000946.2 m: NP_038669.1), EP2 (Accession no. h: NP_000947.2m: NP_032990.1), EP3 (Accession no. h: NP_942009.1 m: NP_032991.1), EP4 (Accession no. h: NP_000949.1 m: NP_000949.1), FP (Accession no. h: NP_000950 m: NP_032992.1), IP (Accession no. h: NP_000951 m: BAA05144), TP (Accession no. h: NP_963998 m: NP_033351.1), DP1 (Accession no. h: NP_000944.1 m: NP_032988.3), and DP2 (CRTH2; Accession no. h: AAR92484.1 m: BAC81437.1). Pairwise sequence alignment determined using CLUSTALW 2.1 Multiple sequence alignment software. Bold shows amino acid homology (%) between same receptor types expressed in human and mice*.

**Table 2:** **Structural, signal transduction, and agonist binding properties of mouse prostaglandin receptors**.

Receptor	Amino acids: human vs. mouse	Signaling	Agonist binding	*K*_i_ (nM)
EP1	402 (human), 405 (mouse)	G_αq_: ↑ [Ca^2+^]_i_	PGE_2_ > PGE_1_ > PGF_2α_	20.00, 36.00, 1300.00
EP2	358 (human), 362 (mouse)	G_αs_: ↑ cAMP	PGE_1_ > PGE_2_	10.00, 12.00
EP3	365–425 (human), 361–365 (mouse)	G_αq_: ↑ [Ca^2+^]_i_	PGE_2_ > PGE_1_	0.68, 0.85
		G_αi_: ↓ cAMP		
EP4	488 (human), 513 (mouse)	G_αs_: ↑ cAMP	PGE_2_ > PGE_1_	1.90, 2.10
FP	359 (human), 366 (mouse)	G_αq_: ↑ [Ca^2+^]_i_	PGF_2α_ > PGD_2_	3.40, 470
IP	386 (human), 417 (mouse)	G_αs_: ↑ cAMP	PGE_1_	33.00
TP	343 (human), 341 (mouse)	G_αq_: ↑ [Ca^2+^]_i_	*[I-BOP]	0.68
DP1	359 (human), 357 (mouse)	G_αs_: ↑ cAMP	PGD_2_	21.00
DP2 (CRTH2)	395 (human), 382 (mouse)	G_αq_: ↑ [Ca^2+^]_i_	PGD_2_	45.00
		G_αi_: ↓ cAMP		

*[I-BOP]: TP receptor agonist; used as a replacement for TxA_2_ due to very short half-life of TxA_2_.

## Prostaglandin Receptors (IP, DP1, EP2, EP4) Activating cAMP Pathway

### PGI_2_-IP receptor

Prostacyclin is a primary prostaglandin produced by endothelial cells and plays an important role in vascular homeostasis (Vane and Botting, 1995). Through the activation of its IP receptor, PGI_2_ induces vasodilation, inhibits platelet aggregation at high concentrations, and proliferates smooth muscle cells via G_αs_-proteins, and increased cAMP levels (Moncada et al., 1977a,b; Falcetti et al., 2010). Due to their tendency for post-translational modifications, the IP receptors are capable of coupling to other signal transduction pathways via G_αq_-protein dependent phosphoinositide turnover and G_αi_-protein dependent inhibition of cAMP (Katsuyama et al., 1994; Hebert et al., 1998; Miggin et al., 2003). IP receptor-mediated vasodilation may also be modulated by the co-activation of EP3 receptors that couple to G_αi_-proteins and therefore further research on role of PGI_2_-IP receptor following stroke is required (Orie and Clapp, 2011).

The role of PGI_2_ in response to neuronal injury or toxicity was measured using rat cortical neuron cultures subjected to hypoxia and glutamate toxicity (Cazevieille et al., 1993, 1994). Following transient ischemia in mice, PGIS and PGI_2_ levels are increased in neurons, macrophages, and microglial cells of the brain (Fang et al., 2006). Increased levels of PGIS and PGI_2_ provided neuroprotection against ischemia indicated by a reduction in infarct volume following adenovirus mediated overexpression of PGIS 72 h after ischemia (Fang et al., 2006). The role of IP receptors in stroke was not determined until recently.

We have also demonstrated the neuroprotective properties of the IP receptor in mouse models of transient middle cerebral artery occlusion (tMCAO) and permanent distal MCAO (pMCAO; Saleem et al., 2010). IP receptor knockout mice showed increased infarct volumes and neurological deficit scores compared with wildtype mice, and pretreatment with a selective IP receptor agonist (beraprost) reduced infarct volumes and deficit scores, confirming the neuroprotective role of IP receptor activation (Saleem et al., 2010). In addition, our group also found that when deleted, IP receptors aggravated hippocampal neuronal loss after bilateral common carotid artery occlusion in mice (Wei et al., 2008). The neuroprotective role of these CNS-specific IP receptor ligands was also demonstrated in monkeys subjected to MCAO (Cui et al., 2006).

Some reports suggest the existence of tissue specific subtypes of IP receptors. It is noteworthy that the activation of IP receptor (IP2) in the CNS prevented oxygen-induced neuronal death and protected CA1 pyramidal neurons against ischemia in gerbils (Takechi et al., 1996; Satoh et al., 1999; Watanabe et al., 1999). In rats subjected to MCAO, the effects of an IP receptor ligand (15-deoxy-TIC) designed to bind the IP2 receptor was found to be significantly neuroprotective when compared with the peripheral type IP receptor ligand, iloprost methylester, administered 24 h after ischemia (Takamatsu et al., 2002).

From evidence supporting the neuroprotective role of IP receptors, we hypothesize that activation of IP receptor could also serve to protect from ICH. The effects of increased IP receptor-dependent activation of cAMP signaling following ICH remain to be fully elucidated. Inversely, because of the vasodilatory properties of PGI_2_, it is possible that the activation of IP receptors would also potentially exacerbate the hemorrhagic transformation and hemorrhagic damage.

### PGD_2_-DP1 receptor

PGD_2_ has both peripheral and central physiologic effects (Whittle et al., 1983; Narumiya and Toda, 1985; Casteleijn et al., 1988; Sturzebecher et al., 1989; Darius et al., 1994; Matsugi et al., 1995; Matsuoka et al., 2000; Angeli et al., 2004). Activated DP1 receptor leads to the activation of the cAMP/PKA pathway and is one of the main cellular mechanism through which DP1 receptors exert their neuroprotective effects (Liang et al., 2005). Activation of the DP1 receptor stimulates adenylyl cyclase, leading to increased levels of cAMP, and decreased platelet aggregation (Hata and Breyer, 2004). PGD_2_ levels are significantly increased under pathologic conditions; however, the effects of PGD_2_ remain dependent on the cell type (Hata and Breyer, 2004; Hatoum et al., 2005). For example, PGD_2_ inhibited TNF-α-induced migration of Langerhans cells, which are involved in cutaneous inflammatory responses by decreasing the infiltration of T helper cells (Angeli et al., 2004). The anti-inflammatory properties of PGD_2_ are primarily associated with DP1 receptors. For example, DP1 receptor activation resulted in decreased allergic and asthma response (Matsuoka et al., 2000; Hammad et al., 2003; Angeli et al., 2004).

In the brain, until recently DP1 receptor related studies were limited to investigations of sleep induction, modulation of body temperature, olfactory function, hormone release, nociception, eye movement, and neuromodulation (Eguchi et al., 1999; Urade and Hayaishi, 1999; Mizoguchi et al., 2001; Hayaishi, 2002; Obal and Krueger, 2003; Angeli et al., 2004; Hata and Breyer, 2004; Gelir et al., 2005; Koch et al., 2005). In addition to expression and function in the brain, the neuroprotective effects of the DP1 receptor was revealed following activation by PGD_2_ protected hippocampal cultures derived from E18 rat embryos against glutamate toxicity (Liang et al., 2005). In a mouse model of cerebral ischemia, we and others have shown that PGD_2_ mediates neuroprotection via the DP1 receptors (Liang et al., 2005; Saleem et al., 2007c). We have also shown that NMDA-induced excitotoxicity and cerebral ischemic brain damage were significantly attenuated by the DP1 receptor-selective agonist BW245C (Ahmad et al., 2010a).

Although the potential neuroprotective effect of DP1 receptors has been reported, the signaling cascade leading to this neuroprotective effect remains unclear. Based on the reports from us and others, it is evident that activation of DP1 receptors leads to increased levels of cAMP/PKA. However, more experiments are required to fully elucidate the role of DP1 receptors in ICH. Based on the role of DP1 receptors in activating the cAMP/PKA pathway, we expect that these receptors could be novel endogenous targets in attenuating ICH. However, the vasodilatory effect of PGD_2_ and DP1 could also pose a higher risk of hemorrhage. Nevertheless, use of pharmacologic and genetic approaches targeting DP1 receptors could provide better insight and might help in protecting the brain from devastating hemorrhagic conditions.

### PGE_2_-EP2 receptor

Among various effects of PGE_2_ many of them have been attributed to its known capacity to stimulate cAMP via EP2 receptors and selective agonist also increase cAMP levels in a concentration-dependent manner with the same potency as PGE_2_ (Narumiya et al., 1999). EP2 receptor-selective agonists increase the cAMP levels in a concentration-dependent manner with the same potency as PGE_2_ (Choi et al., 2001). PGE_2_-stimulated cAMP formation has been shown to be blocked not only by an EP1/EP2 receptor antagonist, AH6809, but also by an inhibitor of adenylyl cyclase, SQ22536 (Fiebich et al., 2001).

In the brain, activation of EP2 receptors by PGE_2_ is involved in long-term synaptic plasticity and cognitive function, where mice deficient in EP2 receptors showed impaired hippocampal synaptogenesis (Sang et al., 2005; Yang et al., 2009). Therefore it is no surprise that brain injuries can affect the expression and function of EP2 receptors. For example, EP2 receptor expression increased in CA1 pyramidal neurons following cerebral ischemia in rats (Choi et al., 2006). However, does this increase in expression of EP2 receptors following brain injury result from its neuroprotective properties when activated? In support of this question, our group and others have shown EP2 receptor-mediated neuroprotection when subjected to glutamate receptor-mediated toxicity and MCAO and that EP2^−/−^ mice has increased damage (Liu et al., 2005; Ahmad et al., 2006b). In a mouse model of focal cerebral ischemia, we have shown that butaprost, an EP2 receptor-selective agonist, provided dose-dependent neuroprotection, whereas deletion of EP2 receptors aggravated ischemic brain damage (Ahmad et al., 2010b). Neuroprotection mediated by EP2 receptor activation occurs by a PKA-dependent mechanism, as demonstrated, against oxidative stress and excitotoxicity (Echeverria et al., 2005).

Additional studies support the mechanism by which PGE_2_ affords neuroprotection through EP2 receptor-associated increases in cAMP, followed by a PKA-dependent pathway (Araki et al., 2000; McCullough et al., 2004; Jiang et al., 2010). In an attempt to discover neuroprotective agents, EP2 receptor agonist conferred neuroprotection in an NMDA receptor-induced excitotoxicity model, strongly reinforcing the notion that EP2 receptor activation by endogenous PGE_2_ in a cell-injury setting is neuroprotective (Jiang et al., 2010). These findings and others suggest the cAMP/PKA pathway is the site of neuroprotection following EP2 receptor activation (Araki et al., 2000; McCullough et al., 2004; Liu et al., 2005). In addition to EP2 receptor-mediated PKA-dependent neuroprotection, cAMP activation independent of PKA may also afford neuroprotection. However, recent studies have reported that PGE_2_ activation of the EP2 receptor had anti-proliferative effects in human gingival fibroblasts and this seems to be mediated by the EP2-cAMP-Epac pathway (Weinberg et al., 2009). Research on cortical neurons showed increased apoptosis through the expression of the Bcl-2 interacting membrane protein Bim and may therefore warrant further research on how Epac regulation may be used as a strategy for the treatment of ICH (Suzuki et al., 2010).

In contrast to previous studies demonstrating the neuroprotective effects of EP2 receptor activation, a recent study showed that when blocked using a selective small molecule EP2 receptor antagonist also significantly reduced neuronal injury in the hippocampus when administered in mice beginning 1 h after termination of pilocarpine-induced status epilepticus (Jiang et al., 2012). This study provides unique insight into the effects of EP2 receptor blockade and may also prove to be a therapeutic strategy for inflammation-related brain injuries such as ICH. Identification of the specific PGE_2_ receptor subtypes involved in acute brain injury following ICH will provide the building blocks for future studies aimed at generating new tools for possible therapeutic interventions. Similarly to EP2 receptors, EP4 receptors have been found to activate the cAMP/PKA pathway; however, EP2 and EP4 receptors have significant differences.

### PGE_2_-EP4 receptor

Like the EP2 receptor, most of the effects PGE_2_ mediated activation of EP4 receptor is due to cAMP signaling; however, there is a subtle difference between their abilities to stimulate cAMP. Using COS cells expressing both EP2 and EP4 receptors, PGE_2_ stimulation produced ∼11- and ∼8-fold increase in cAMP, respectively (Honda et al., 1993; Regan et al., 1994). EP4 receptor-mediated weaker coupling to G_αs_-proteins suggests cAMP plays a less important role in EP4 receptor signaling compared to EP2 receptors (Fujino et al., 2002, 2005). Regardless of the differences in the formation of intracellular cAMP, EP4 receptor had a greater affinity for PGE_2_ than the EP2 receptor (Fujino et al., 2003). Compared to EP2, EP4 also has a longer C-terminus, which has been implicated in agonist-induced desensitization and internalization (Nishigaki et al., 1996; Bastepe and Ashby, 1999; Desai et al., 2000). Despite the low levels of intracellular cAMP, when activated following injury, the EP4 receptor has proven to have neuroprotective properties.

It is well documented that EP4 receptor activation has anti-inflammatory effects (Narumiya, 2009; Shi et al., 2010; Tang et al., 2012). Also, in the same study, conditional inactivation of EP4 receptor in neurons and endothelial cells increased infarct size in mice subjected to MCAO. In addition to its neuroprotective effects, EP4 receptor activation blocked LPS-induced pro-inflammatory gene expression in the brain tissue of mice. Additionally, an *in vivo* study by the same group, demonstrated that conditional deletion of EP4 receptor in macrophages and microglia increased lipid peroxidation and pro-inflammatory gene expression in the brain (Shi et al., 2010). We have shown that β-amyloid-induced (Aβ42) toxicity is minimized by EP4 receptor activation (Echeverria et al., 2005). Similarly, following acute striatal excitotoxicity, activation of EP4 receptor protected the brain (Ahmad et al., 2005).

In addition to their role in neuroprotection, endothelial cells expressing EP4 receptor are involved in vasodilation due to the direct activation of endothelial NOS, thus their role in the relaxation of smooth muscles (Dumont et al., 1999). Because EP4 receptor signaling is more complex (involving not only G_αs_-protein mediated increase in cAMP but also coupling to pertussis toxin-sensitive G_αi_-proteins and β-arrestin mediated effects), the role of EP4 receptors in ICH may be more dynamic than in ischemic stroke (Penn et al., 2001; Fujino and Regan, 2006).

Due to the role of EP4 receptors in vasodilation, we hypothesize that activation of EP4 receptors may play an important role in the control of cerebral blood flow, and thus may represent a novel target for the prevention or treatment of cerebral ischemia. In ICH, the endothelium and cerebral vasculature are compromised by potential brain injury with the expansion of blood away from the region of hematoma. To date, the exploration of EP4 receptors as a target to protect or rescue neurons from blood components after ICH remains to be studied. As mentioned, the effects of PGE_2_ are vast and beyond the scope of this review; however, the varied effects of the PGE_2_ may be attributed to the receptors onto which it binds and activates. For example, PGE_2_ when bound to its receptors cannot only stimulate cAMP signaling but also calcium signaling by binding to EP1 and FP receptors.

## Prostaglandin Receptors (EP1 and FP) Activating Calcium Pathway

### PGE_2_-EP1 receptor

When activated, EP1 receptors couple to G_αq_-proteins, resulting in increased phosphatidyl inositol hydrolysis and elevation of intracellular Ca^2+^. EP1 receptor activation by PGE_2_ leads to increased Ca^2+^ signaling and subsequent neurotoxicity (Kandasamy and Hunt, 1990; Kawano et al., 2006). EP1 receptor-mediated neuronal toxicity was found to be normalized to basal levels following either blockade or deletion of EP1 receptors, and improved function of the Na^+^/Ca^2+^ exchanger (Kawano et al., 2006). Glial-derived neurotrophic factor (GDNF) therapy has been shown to be beneficial in treating Parkinson’s disease when EP1 receptor is blocked or ablated, resulting in a 60% enhancement in GDNF, which suggests that selective inhibition of EP1 receptor signaling might be a means to augment GDNF secretion in diseased regions of the brain (Li et al., 2012).

Due to decreased calcium signaling, pharmacological blockade of EP1 receptor could make good therapeutic targets against brain injury (Gendron et al., 2005). Therefore, using EP1^−/−^ mice and selective EP1 receptor antagonist, we and others have found that EP1 receptors contribute to excitotoxicity following focal cerebral ischemia (Ahmad et al., 2006a, 2008; Saleem et al., 2007b; Abe et al., 2009). Using a neonate model of hypoxic-ischemia encephalopathy, EP1 receptor blockade with a selective antagonist, SC-51089, and co-activation of EP2–4 receptors, cerebral injury was reduced 24 h after injury (Taniguchi et al., 2011b). The role of Ca^2+^ signaling in neuronal activity and death following ICH is unknown; however, current data suggests that increased Ca^2+^ signaling contributes to increased cerebral vasospasm commonly seen in SAH (Kikkawa et al., 2010; Koide et al., 2011). Neuroprotection following the blockade of EP1 receptor has been found to involve the PTEN/AKT survival pathway following ischemic stroke (Zhou et al., 2008; Abe et al., 2009). Relating the blockade of EP1 receptor to neuroprotection *in vivo* may be complicated by the role of various cells types; for example, neuroprotection via the blockade of EP1 receptor was reduced by the presence of microglia in NMDA stimulated neuron-glial cultures (Carlson et al., 2009). The role of EP1 receptor in ICH is unknown. However, one study showed increased expression of EP1 receptors following hemorrhage in splenic macrophages from male mice (Stapleton et al., 2004).

Data accumulating thus far from ischemic brain injuries and recent data from our group on ICH suggests that activation of EP1 receptor might lead to different outcomes in different ICH models. Models of ICH at different time points, using both EP1 receptor knockout and pharmacological approaches to elucidate the role of EP1 receptor would increase our *in vivo* knowledge of EP1 receptors in ICH. Therefore, we recommend further detailed investigations into the potentialities of EP1 receptor as a therapeutic target.

### PGF_2α_-FP receptor

The amino acid sequence of FP receptor has a high sequence homology with that of the PGE_2_-EP1 receptor, and shares the same phylogenetic branch with EP1 receptor (Toh et al., 1995). Despite the abundance of arachidonic acid in the brain, the function of its metabolite, PGF_2α_, is poorly understood. However, PGF_2α_ is known to play a significant role in the initiation of parturition, renal function, control of cerebral blood flow, and intraocular pressure principally by an increase in uveoscleral outflow of aqueous humor, autoregulation in newborn piglets, contraction of arteries, and myocardial dysfunction (Chemtob et al., 1990; Sugimoto et al., 1997; Takayama et al., 2005; Jovanovic et al., 2006; Hao and Breyer, 2007). Pathological conditions in humans influence PGF_2α_ levels in cerebrospinal fluid, where elevated levels of PGF_2α_ were measured following epilepsy, meningtitis, brain injury, and stroke (Wolfe and Mamer, 1975; Kostic et al., 1984). Interestingly, elevated levels of PGF_2α_ in the CSF of patients following acute cerebral ischemia did not correlate with the degree of neurological deficit (Kostic et al., 1984).

Activation of FP receptor initiates several events, including stimulation of the phospholipase C/IP_3_R/Ca^2+^ signaling pathway (Heaslip and Sickels, 1989; Abramovitz et al., 1994; Knock et al., 2005). FP receptor activation causes the release of Inositol 1,4,5-triphosphate and diacylglycerol in turn activating the calcium-calmodulin-CaMK-II pathway, which may be associated with FP receptor-mediated excitotoxicity following transient focal brain ischemia (Narumiya et al., 1999; Saleem et al., 2009a). Using FP^−/−^ mice we have shown that FP receptor is involved in the enhancement of cerebral ischemia and excitotoxic brain injury and may mitigate the effects of ischemic stroke brain injury (Saleem et al., 2009a). Recently, we have also demonstrated that FP receptor blockade and knockout protects against ischemic stroke in mice and oxygen-glucose deprivation-induced cell death in slice cultures (Kim et al., 2012).

Although FP receptor has been implicated in ischemic stroke, the roles of PGF_2α_ and FP receptor have not been studied in the pathogenesis of ICH. Because FP receptor regulates [Ca^2+^]_i_ levels, we propose that the activation subsequent to injury contributes to excitotoxicity and hemorrhagic brain damage. Therefore, blockade of FP receptor would be beneficial for the treatment of ischemic stroke.

## Prostaglandin Receptors (EP3, TP, DP2) Activating Both cAMP and/or Calcium Pathway

### PGE_2_-EP3 receptor

Because of its coupling to several G-proteins the EP3 receptor has various biological properties. EP3 is important in a number of physiological functions including vasoconstriction of the pulmonary arteries, growth inhibition of keratinocytes, and inhibition of aromatase activity in breast fibroblasts (Qian et al., 1994; Konger et al., 1998, 2005; Richards and Brueggemeier, 2003). The EP3 receptor has multiple splice variants that differ in both their C-terminal tails and signaling pathways (Kotani et al., 1995). The activation of the human EP3 subtypes EP3_I_, EP3_II_, EP3_III_, EP3_IV_, EP3_e_, and EP3_f_ isoforms have been shown to inhibit cAMP production, whereas stimulation of EP3_I_, EP3_II_, and EP3_III_ increase IP_3_/[Ca^2+^]_i_ (Kotani et al., 1995; Schmid et al., 1995). The EP3 receptor isoforms expressed in mice (α, β, and Γ) contain carboxyl tails of 30, 26, and 29 amino acids that modulate signal transduction (Irie et al., 1994). In this context, EP3_α_ and EP3_β_ receptors couple to G_αi_-proteins and inhibit cAMP, whereas the EP3_γ_ couples to G_αs_-proteins in addition to G_αi_-proteins and evokes cAMP production (Sugimoto et al., 1993). In neurological diseases such as stroke, the function of EP3 receptor so far is not firmly defined; this may be in part due to the variants of the EP3 receptor subtypes.

When activated with selective EP3 receptor agonist ONO-AE-248, our group recorded a dose-dependent increase in infarct volume after MCAO in mice (Ahmad et al., 2007). Recently, the proposed mechanism of EP3 receptor-mediated neuronal death in stroke has been implicated by the glutamate-dependent increase in mPGES-1 activity, which in turn increases EP3 receptor activation and activation of Rho and/or G_αi_ proteins signaling (Ikeda-Matsuo et al., 2010). In contrast to EP3 activation, we have shown that the deletion of EP3 receptor results in decreased infarct volume 48 h after ischemia (Saleem et al., 2009b). Another group showed that deletion of the EP3 receptor did not alter infarct volume or behavior 24 h after ischemia (Li et al., 2008). The discrepancy in results could be due to differences in time points post-MCAO used to measure infarct volume and the age of the mice used in both studies. Also, recently it has been shown that when EP3 receptors are deleted, damage is done to the blood-brain barrier, activation of microglia and infiltration of neutrophils into the ischemic cortex are reduced, and the underlying neuroprotection mediated by EP3 receptor deletion may be due to decreased inflammation and apoptotic signaling (Ikeda-Matsuo et al., 2011).

No current study has correlated the activation of EP3 receptors in ICH. Three isoforms of the EP3 receptor are expressed in mice; consequently, the EP3 receptor role in ICH may prove to be different than its role in ischemic stroke because different cell types may not only express different levels of EP3 receptors, but their functions may be different after ICH. Furthermore, due to the differences between isoforms, it is possible that the dose and time lapse in administration of EP3 receptor drug treatments post-stroke could regulate the final outcomes. Therefore, use of conditional EP3 receptor transgenic animals or a therapeutically selective agonist/antagonist for these EP3 receptor isoforms could be beneficial in discerning the role that each of these isoforms plays following ICH.

### TxA_2_-TP receptor

TxA_2_-TP receptor activity is coupled with G_αs_-, G_αq_-, G_αi_-, and G_α12/13/15/16_-proteins (Shenker et al., 1991; Laugwitz et al., 1996; Muck et al., 1998). These G-proteins in turn regulate several effectors including phospholipase C, adenylyl cyclase, cAMP, guanine nucleotide exchange factor of the small G-protein Rho, and intracellular calcium (Kozasa et al., 1998). In humans there are two isoforms of TP receptors, TPα (placental/platelet) and TPβ (endothelial), which account for some of the differences in intracellular signaling after activation of TP receptors. These isoforms differ in length and sequence at the C-terminal distal to the last amino acid (Arg328) and are expressed in most tissues including platelets, placenta, vascular smooth muscle, brain, small intestine, and thymus (Colman, 1991; Hirata et al., 1991; Namba et al., 1992; Ushikubi et al., 1993; Raychowdhury et al., 1994; Miggin and Kinsella, 1998). Despite the relatively limited physiological role of TPβ receptors, their activation results in pertussis toxin-sensitive inhibition of cAMP (Namba et al., 1992; Hirata et al., 1996). In mice, no TP receptor isoforms exist; therefore, TP receptors in mice are considered to be similar to human TPα (74% identical, see Table 2 and Figure 1) and when activated, mediate the increase in cAMP following I-BOP (a TP receptor agonist) treatment.

Due to the isoforms, TP receptor signaling in humans varies with tissue type. For example, in platelets, TP receptor coupling to G_αq_-proteins results in increased [Ca^2+^]_i_ (Hirata et al., 1994b). In addition, platelet activation may also occur due to theG_α12/13_-protein pathways (Offermanns et al., 1996). In the CNS, hippocampal TP receptor plays a functional role in both neuronal excitability and synaptic transmission (Schwartz-Bloom et al., 1996). Activation of presynaptic TP receptor results in increased glutamate release, and postsynaptic activation of TP receptor results in the inhibition of synaptic transmission, suggesting that TP receptor plays different roles based on localization (Hsu and Kan, 1996). Moreover, activation of TP receptor dose-dependently suppressed whole-cell Ca^2+^ currents in rat CA1 neurons (Hsu et al., 1996).

TP receptors play an important role in the development of cerebral ischemia and mediate vascular proliferation and contraction (De Clerck and Janssen, 1990). Following SAH, mRNA and protein levels of TP receptors were elevated in cerebral arteries and smooth muscle cells, respectively (Ansar et al., 2010). Elevated levels of TxA_2_ in CSF have been reported in patients after SAH and ICH (Pickard et al., 1994; van Kooten et al., 1999b). TxA_2_ mediated activation of TP receptors expressed on platelets surrounding the region of the brain (cortex and striatum) has been linked to exacerbating ICH injury (Kong et al., 1991; van Kooten et al., 1999a; Saloheimo et al., 2005; Yalcin et al., 2005). Due to the expression and distribution of TP receptor in the brain, TP receptors could be considered an ideal therapeutic target for the treatment of ICH. Platelet activation is often a reflection of vascular risk factors, diffused atherosclerotic lesions, or the extent of damage caused by stroke. Terutroban, a selective TP receptor antagonist (*K*_i_ = 0.82 nM) used as an antithrombotic agent, could also be used to prevent atherothrombosis and ischemic stroke (Chamorro, 2009). Using a spontaneous hypertensive stroke-prone rat model, terutroban increased the survival rate by reducing systemic inflammation, thus promoting the TP receptor antagonists therapeutic intervention for stroke as a possible antithrombotic agent in human compared to aspirin alone (Gelosa et al., 2010). However, in a recent human study, no significant differences in outcomes were recorded between terutroban and aspirin in patients with cerebral ischemia (Bousser et al., 2011). Several receptors are upregulated following ischemia and the mulifactorial nature of ischemia may explain the lack of effect of many substances tested in clinical trials. These accumulating data warrant further examination of the therapeutic potential of TP receptors and their selective ligands to fully elucidate their role in ICH.

### PGD_2_-DP2 (or CRTH2) receptor

One of two PGD_2_ receptors, DP2 receptor, is the most recently discovered. DP2 receptor was initially cloned as an orphan receptor and later identified as T helper cell type 2 cells (Th2), specific surface PGD_2_ receptor (Nagata et al., 1999a,b; Hirai et al., 2001). The DP2 receptor is distinguished by their similarity to chemoattractant receptors and thus, they are also known as chemoattractant receptor-homologous molecules expressed on Th2 cells (CRTH2; Monneret et al., 2001). The CRTH2 receptors are also known as DP2 receptor based on their similar binding affinity to that of DP1 toward PGD2, although the DP1 and DP2 receptors do not share structural homology (Hirai et al., 2001). Similar to many chemoattractant receptors, the DP2 receptor is coupled to G_αi_-proteins, leading to the inhibition of cAMP and increase in Ca^2+^ in a variety of cell types (Sawyer et al., 2002). Given the role of the DP2 receptor in inflammatory diseases such as asthma, blocking of DP2 receptor represents a novel therapeutic approach for the treatment of such conditions.

Despite its expression in the spinal cord and its anti-inflammatory effect as an activator of peroxisome proliferator-activated receptors, little is known about the role of DP2 receptors after stroke (Genovese et al., 2008; Grill et al., 2008; Morgenweck et al., 2010). Based on the role of the DP2 receptor in cAMP inhibition and Ca^2+^ activation, we hypothesize that activation of this receptor could lead to aggravated brain damage and its inhibition could lead to better functional and anatomical outcomes after ICH. Moreover, blockade of this receptor could result in more availability of PGD_2_ to DP1 receptor, which could then lead to increased cAMP level and subsequent neuroprotection as we have previously reported (Saleem et al., 2007c; Ahmad et al., 2010a). We hypothesize that blockade of DP2 receptor following ICH will result in greater injury based on the increased injury we observed with DP1^−/−^ mice following ischemic stroke and acute NMDA-induced excitotoxicity due to similar binding affinity to DP1 receptor, DP2 receptors would have little therapeutic potential in ICH (Ahmad et al., 2010a).

## Conclusion

Intracerebral hemorrhage accounts for 13% of all strokes in the United States each year. Following ICH, extravasated blood accumulates and compresses the surrounding brain tissue and treatment is primarily supportive with poor clinical outcome. To improve the outcome of ICH patients, a better understanding of the pathogenesis of ICH-induced brain injury is needed. We and others have identified prostaglandin receptors as potential therapeutic agents against ischemic stroke. Prostaglandins and their receptors regulate many physiological, inflammatory, and immunological processes. In the CNS, the role of prostaglandin receptors has been essentially first elucidated by gene-deletion studies and evidence suggests these receptors are a therapeutic target for the treatment of stroke. However, the therapeutic potential of prostaglandin receptors in ICH remains to be elucidated. Understanding the integration of a network of prostaglandin receptor signaling would improve our knowledge of these putative therapeutic targets for the effective treatment of neurological diseases. Evidence supports that prostaglandin receptors play a cooperative and/or sequential role in ICH-mediated inflammation and neurotoxicity. Intracellular signaling pathways activated by prostaglandin receptors in ICH are likely to be different than in ischemic stroke. However, we hypothesize that evolutionary regulated prostaglandin receptor signaling will be conserved and therefore this group of receptors, found to have a given role in ischemia may also have a unique role in hemorrhagic stroke. However it remains to be determined whether the effect of these receptors will be similar. Also, identification of a multitude of intracellular protein interactions with prostaglandin receptors may prove to regulate their neuronal expression, selectivity toward ligands, and crosstalk with cytokines, chemokines, and neurotransmitters. Studying these interactions and the cell-specific functions will help map a detailed network of signaling that would be necessary for an injury-induced cellular response. The discovery of a prostaglandin receptor specific intracellular response may enable us to navigate a complex pathway leading to the discovery of beneficial drugs for the treatment of hemorrhagic stroke.

## Conflict of Interest Statement

The authors declare that the research was conducted in the absence of any commercial or financial relationships that could be construed as a potential conflict of interest.

## Abbreviations

15-deoxy-TIC, 15-deoxy-(16-*m*-tolyl)-17,18,19,20 tetranorisocarbacyclin methyl ester; AKT, serine/threonine-specific protein kinase family; BP, blood pressure; BW245C, (4S)-(3-[(3R,S)-3-cyclohexyl-3-hydroxypropyl]-2,5-dioxo)-4-imidazolidineheptanoic acid; BWA868C, 3-[(2-cyclohexyl-2-hydroxyethyl)amino]-2,5-dioxo-1-(phenylmethyl)-4-imidazolidineheptanoic acid; CA1 pyramidal neurons, *Cornu Ammonis*-1 pyramidal neurons; CaMK, Ca^2+^/calmodulin-dependent protein kinase; cAMP, cyclic-adenosine monophosphate; COX, cyclooxygenase; CRTH2, chemoattractant receptor expressed Th2 lymphocytes; CSF, cerebrospinal fluid; Epac, exchange protein directly activated by cAMP; Gαi-protein, guanine nucleotide-binding protein (inhibitory); Gαq-protein, guanine nucleotide-binding protein (stimulatory/inhibitory); Gαs-protein, guanine nucleotide-binding protein (stimulatory); I-BOP, (Z)-7-[(1S,4R,5R,6R)-5-[(E,3R)-3-hydroxy-4-(4-iodophenoxy)but-1-enyl]-7-oxabicyclo[2.2.1]heptan-6-yl]hept-5-enoic acid; ICH, intracerebral hemorrhage; ICP, intracranial pressure; IP_3_R, inositol trisphosphate receptor; MCAO, middle cerebral artery occlusion; MK-0524, 2-[(3R)-4-[(4-chlorophenyl)methyl]-7-fluoro-5-methylsulfonyl-2,3-dihydro-1H-cyclopenta[b]indol-3-yl]acetic acid; NMDA, *N*-methyl-d-aspartic acid; NOS, nitric oxide synthase; ONO-AE-248, (Z)-7-[(1R,2R,3R)-3-methoxy-2-[(E,3S)-3-methoxyoct-1-enyl]-5-oxocyclopentyl]hept-5-enoic acid; PGDS, prostaglandin D synthase; PGES, prostaglandin E synthase; PGFS, prostaglandin F synthase; PGIS, prostaglandin I synthase; PKA, protein kinase A; PTEN, phosphatase and tensin homolog; SAH, subarachnoid hemorrhage; TS-022, 4-[(1R,2S,3R,5R)-5-Chloro-2-((*S*)-3-cyclohexyl-3-hydroxyprop-1-ynyl)-3-hydroxycyclopentyl]butylthio acetic acid monohydrate; TxS, thromboxane synthase.
